# False diagnosis of recurrent thyroid carcinoma: the importance of testing for heterophile antibodies

**DOI:** 10.20945/2359-4292-2023-0115

**Published:** 2024-03-15

**Authors:** Leila Guastapaglia, Maria Izabel Chiamolera, José Viana Lima, Claudia Maria De Francischi Ferrer, Luciana Godoy Viana, Claudia Veiga Chang, Raquel Andrade Siqueira, Rui Monteiro Barros Maciel, José Gilberto Henriques Vieira, Rosa Paula Mello Biscolla

**Affiliations:** 1 Universidade Federal de São Paulo Departamento de Medicina Divisão de Endocrinologia São Paulo SP Brasil Centro de Doenças da Tireoide e Laboratório de Endocrinologia Molecular e Translacional, Divisão de Endocrinologia, Departamento de Medicina, Escola Paulista de Medicina, Universidade Federal de São Paulo, São Paulo, SP, Brasil; 2 Grupo Fleury São Paulo SP Brasil Grupo Fleury, São Paulo, SP, Brasil; 3 Faculdade do Instituto Superior de Medicina Divisão de Endocrinologia São Paulo SP Brasil Divisão de Endocrinologia, Faculdade do Instituto Superior de Medicina (ISMD), São Paulo, SP, Brasil; 4 Hospital Geral de Goiânia Divisão de Endocrinologia Goiânia GO Brasil Divisão de Endocrinologia, Hospital Geral de Goiânia (Hospital Alberto Rassi), Goiânia, GO, Brasil

## Abstract

Thyroglobulin (Tg) levels are important to predict recurrence in differentiated thyroid cancer patients. However, false-positive results can hence the request of unnecessary tests and treatments. We reported two cases of interference in thyroglobulin measurement and the workup to investigate them. Both patients achieved an excellent response to therapy after total thyroidectomy and one patient had also received radioiodine treatment. During the follow-up, Tg levels increased and there was no evidence of recurrent disease in the imaging studies. The Tg levels by the Access platform were positive but the results by Elecsys platform and LC-MS/MS were undetectable, leading to the hypothesis of heterophile antibodies (HAbs) interference. The possibility of HAbs interference must be considered when the Tg levels do not fit in the clinical picture. The measurement of Tg by another immunoassay or by LC-MS/MS may be useful in these situations.

## INTRODUCTION

The measurement of thyroglobulin (Tg) is an important tool to predict disease persistence and recurrence in differentiated thyroid carcinoma (DTC) patients after treatment with total thyroidectomy and radioiodine ablation. Undetectable Tg measured by immunometric assays (IMAs) with high functional sensitivity (<0.1 ng/mL) and negative imaging studies indicate an excellent response to treatment (1,2). Although the IMAs used in the laboratory routinely are sensitive enough to detect early disease recurrence during follow-up, some pitfalls still exist in clinical practice, including the following: the presence of thyroglobulin autoantibodies (TgAbs) in 15%-20% of DTC patients, heterophile antibodies and tumor production of anomalous Tg isoforms. In these situations, Tg becomes an unreliable tumor marker (3–6). TgAbs are the most important confounder affecting the accurate determination of Tg by IMAs and they can cause falsely low or undetectable readings (5,7). On the other hand, a positive result is generally assumed to be real and not usually suspected for interference, which leads to the misdiagnosis of an incomplete response to therapy and hence the request for unnecessary tests and treatments (8–10).

Heterophile antibodies (HAbs) can bind to the antibodies employed in IMAs, leading to false-positive (more frequently) or false-negative results (11). In the literature, the reported prevalence of HAbs interference in thyroglobulin assays ranges from 0.4% to 3% (3,11–13). The presence of HAbs should be considered in DTC patients whose Tg measurements are not consistent with clinical and imaging findings. In those patients, the workup of samples with suspected HAbs includes Tg measurement after serial dilutions, pretreatment with HAbs blocking reagents/tubes and reassaying with different immunoassay platform or methodology (14,15). The measurement by liquid chromatography–tandem mass spectrometry (LC-MS/MS) can be useful in these situations (9,16).

We report two cases of HAbs interference in thyroglobulin measurements leading to unnecessary investigation and the workup used to find out this interference.

## PATIENTS

**Patient #1:** A 41-year-old woman was assessed for a persistently high serum Tg level after total thyroidectomy. The histology showed a 1.6 cm follicular variant of papillary carcinoma with minimal extra thyroid extension, and the patient was classified as having an intermediate risk of recurrence (American Thyroid Association- ATA). She received 150 mCi of radioactive iodine (RAI). The posttherapy whole-body scan (WBS) showed uptake in the thyroid bed, and the Tg level was 10.7 ng/mL (TSH 82 mUI/L), with negative TgAbs. After the initial treatment, undetectable Tg levels under levothyroxine therapy (Tg-LT4) and after stimulation with recombinant human TSH (rhTSH) were observed and, the patient was classified as having an excellent response to therapy. One year later, the Tg-LT4 level increased to 7.5 ng/mL, confirmed in a second measurement (5.3 ng/mL). She underwent a neck ultrasound (US), WBS and ^18^FDG PET/CT scan that showed no uptake or suspicious images, and the Tg level stimulated by rhTSH (Tg-rhTSH) was 7.9 ng/mL. Since the imaging studies were negative, she was reclassified as having a biochemical incomplete response to therapy. Nine months later, the Tg-LT4 level increased to 10.6 ng/mL, and she underwent another WBS and ^18^FDG PET/CT scan, both negative; the Tg-rhTSH level was 14.8 ng/mL. The high serum Tg level prompted a further evaluation with abdominal MRI that did not show suspicious images. Two lymph nodes were identified in the neck by US, and guided biopsy showed lymphoid cells and undetectable Tg in the needle washout. The patient was referred to the laboratory to evaluate for interference in the Tg measurements. The Tg level measured by the Access platform (Beckman Coulter, Fullerton, CA, USA) was 30 ng/mL, and the serial dilutions were linear (Table 1). We performed Tg retesting using another IMA platform (Elecsys II, Roche) and the LC-MS/MS from Mayo Medical Laboratories (functional sensitivity < 0.5 ng/mL), and both measurements were undetectable.

**Table 1:** Access Tg concentrations neat and diluted, Elecsys Tg II Roche and LC-MS/MS Tg concentrations

Patient	Access Tg, ng/mL				
	Neat	x2*	x5*	Elecsys Tg II Roche, ng/mL	LC-MS/MS Tg, ng/mL
1	30	29	28	<0.1	<0.5
2	8.9	9	10	<0.1	<0.5

* x2 and x5: 1:2 and 1:5 dilutions from neat, respectively.

**Patient #2:** A 36-year-old woman underwent total thyroidectomy for papillary thyroid microcarcinoma. She was stratified as having a low risk of recurrence by the ATA consensus and was not treated with adjuvant RAI therapy. During the follow-up, she had both undetectable Tg-LT4 and Tg-rhTSH levels and a negative WBS. After nine years, the Tg-LT4 level increased to 17 ng/mL. She was evaluated with neck US and WBS, both negative, and the Tg-rhTSH level was 12 ng/mL. The negative results from the WBS and the paradoxical decrease in the Tg-rhTSH level compared with the Tg-LT4 level raised the suspicion of assay interference. The Tg level measured by the Access platform was 8.9 ng/mL, and the serial dilutions showed linearity. The Tg levels measured by the Elecsys platform and by LC-MS/MS were undetectable (Table 1).

## DISCUSSION

The follow-up of DTC patients is based on the measurements of Tg and TgAbs and neck ultrasound (1). False-positive Tg measurements can lead to unnecessary tests and treatments, which may include RAI and even invasive procedures (9,10). A false-positive measurement of Tg should be always suspected when the Tg level does not fit the clinical picture (9,17).

In the two cases reported, the low probability of tumor recurrence, the increase in Tg levels after achieving an excellent response to therapy, the absence of disease in imaging studies and the lack of increment in the Tg-rhTSH level compared with the Tg-LT4 level prompted the investigation for interferents. We initially proceeded the investigation with Tg measurements in the Access platform after dilutions of 1:2 and 1:5 from the neat sample. In both cases, the dilution curve was unexpectedly linear. The next step was to retest the samples by a different immunoassay platform (Roche Elecsys II) and by LC-MS/MS which resulted in undetectable Tg levels, confirming HAbs interference.

**Figure 1:**
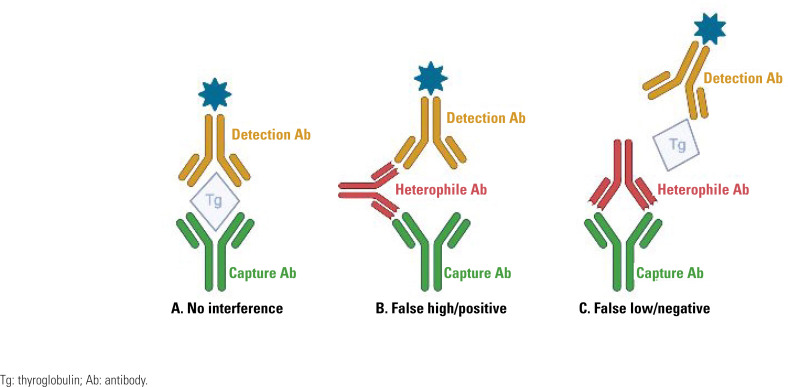
(**A**) Schematic depiction of immunometric assays for the measurement of Tg without interference; (**B**) Schematic depiction of false-positive/false high interference for heterophile antibody; (**C**) Schematic depiction of false-negative/false low interference for heterophile antibody.

The term heterophile antibody is used to describe any antibody that may cause false results in immunoassays by binding to the assay antibodies. HAbs generally bind to both the capture and detection antibodies in sandwich assays, forming a bridge that simulates the presence of an analyte leading to false-positive results in its absence or falsely increased measurements when the analyte is present (Figure 1B). HAbs may also bind to the capture and/or detection antibody, preventing the binding of the analyte and the formation of the antigen-antibody sandwich complex, causing false-negative or falsely low results (Figure 1C). This last situation occurs less frequently and may be more challenging to detect (3,11,13,18).

An investigation of HAbs includes a) serial dilutions to evaluate linearity; b) pretreatment with heterophile-blocking reagents/tubes; c) to reassay with a different immunoassay, since samples that show interference in one particular assay may not present the same problem using an assay from another manufacturer; or d) Tg measurement by another method, such as LC-MS/MS (14,15,19). The presence of HAbs should be considered when the dilution curve is nonlinear (results deviate more than +/- 20% from the undiluted sample concentration after correction for the dilution factors), pretreatment with HAb-blocking reagents substantially alters the results or when the values from other assays are not similarly increased (3). Although the first steps can generally detect the presence of HAbs, they frequently fail to provide accurate Tg concentration values (9,20).

Recently, the measurement of Tg by LC-MS/MS was described as useful in these situations. The LC-MS/MS assay is based on the direct quantification of Tg after using trypsin to cleave proteins. In this process, large proteins, such as Tg, are broken into small peptides, which enables their measurement by mass spectrometry. Then, a specific Tg peptide is separated by liquid chromatography and finally detected using mass spectrometry. In the same process in which Tg is cleaved, as a secondary benefit, there is also the digestion of antibodies, eliminating HAbs as interferers (20). Netzel and cols. (16) and Barbesino and cols. (9) reported cases in which the traditional workup failed to provide accurate Tg results, although it was able to detect HAbs interference in most cases. These authors proposed that samples suspected for HAbs interference should always be initially evaluated by LC-MS/MS. Although the results obtained by LC-MS/MS measurements are precise, this method is not available in many centers.

There is not a consensus in the literature on the frequency of HAbs interference in immunoassays. The rates of interference may also depend on the type of assay used (18). Preissner and cols. (3) reported a rate of 3% false-positive results in 1106 serum Tg samples analyzed by the Access platform and Giovanella and cols. (11) showed that HAbs interference in Tg measurements could be found in 1% of the samples analyzed by the Immulite platform. They also reported some falsely lowered Tg values, although false higher concentrations were the majority. Verburg and cols. (12) reported a similar rate of interference in the Tg measurements on the BRAHMS platform (2/201 in DTC samples, 0/52 in controls). In our cases, the interference was only observed on the Access platform and not on the Elecsys II platform.

The two cases reported in this study show the importance of investigating HAbs in patients whose Tg levels are not compatible with the ongoing follow-up before exposing them to unnecessary tests and treatments. It is worth noting that a positive result is generally assumed to be real and not usually suspected for interferents, which leads to the misdiagnosis of an incomplete response to therapy. In the cases we reported, measurements after serial dilutions did not show the interference of HAbs. On the other hand, the measurement using a different IMA platform or LC-MS/MS was essential for the workup of HAbs interference in those patients.
